# Spatial distribution and movement of Atlantic tarpon (*Megalops atlanticus*) in the northern Gulf of Mexico

**DOI:** 10.1371/journal.pone.0298394

**Published:** 2024-03-07

**Authors:** Shane A. Stephens, Michael A. Dance, Michelle Zapp Sluis, Richard J. Kline, Matthew K. Streich, Gregory W. Stunz, Aaron J. Adams, R. J. David Wells, Jay R. Rooker

**Affiliations:** 1 Department of Marine Biology, Texas A & M University at Galveston, Galveston, Texas, United States of America; 2 Department of Oceanography and Coastal Sciences, Louisiana State University, Baton Rouge, Louisiana, United States of America; 3 School of Earth, Environmental, and Marine Sciences, University of Texas Rio Grande Valley, Brownsville, Texas, United States of America; 4 Harte Research Institute of Gulf of Mexico Studies, Texas A&M University-Corpus Christi, Corpus Christi, Texas, United States of America; 5 Bonefish & Tarpon Trust, Miami, Florida, United States of America; 6 Florida Atlantic University Harbor Branch Oceanographic Institute, Fort Pierce, FL, United States of America; Universidade de Aveiro, PORTUGAL

## Abstract

Atlantic tarpon (*Megalops atlanticus*) are capable of long-distance migrations (hundreds of kilometers) but also exhibit resident behaviors in estuarine and coastal habitats. The aim of this study was to characterize the spatial distribution of juvenile tarpon and identify migration pathways of adult tarpon in the northern Gulf of Mexico. Spatial distribution of juvenile tarpon was investigated using gillnet data collected by Texas Parks and Wildlife Department (TPWD) over the past four decades. Generalized additive models (GAMs) indicated that salinity and water temperature played a significant role in tarpon presence, with tarpon occurrences peaking in the fall and increasing over the past four decades in this region. Adult tarpon caught off Texas (n = 40) and Louisiana (n = 4) were tagged with acoustic transmitters to characterize spatial and temporal trends in their movements and migrations. Of the 44 acoustic transmitters deployed, 18 of the individuals were detected (n = 16 west of the Mississippi River Delta and n = 2 east of the Mississippi River Delta). Tarpon tagged west of the Mississippi River Delta off Texas migrated south in the fall and winter into areas of south Texas and potentially into Mexico, while individuals tagged east of the delta migrated into Florida during the same time period, suggesting the presence of two unique migratory contingents or subpopulations in this region. An improved understanding of the habitat requirements and migratory patterns of tarpon inhabiting the Gulf of Mexico is critically needed by resource managers to assess the vulnerability of each contingent to fishing pressure, and this information will guide multi-state and multi-national conservation efforts to rebuild and sustain tarpon populations.

## Introduction

Atlantic tarpon (*Megalops atlanticus*) are popular and highly targeted gamefish in coastal waters of the Atlantic Ocean [1]. Their large size and acrobatic fight attract fishermen from all over the world, contributing to a multibillion-dollar recreational fishing industry [1–3]. Tarpon occur from Bermuda to Brazil in the western Atlantic Ocean [4]. This species is capable of long-distance migrations, often displaying movements over hundreds of kilometers; however, resident behaviors are also evident with some individuals showing limited movement by remaining in certain estuarine and coastal areas [2]. Well-developed seasonal migration patterns have been reported for tarpon with individuals often moving to higher latitudes in the late spring and early summer, and then migrating back to lower latitudes in the late summer and early fall [2, 5, 6]. Although our understanding of tarpon migrations has improved in recent years, more spatially resolved data on their distribution, habitat requirements, and movements in certain geographic regions are lacking, compromising the ability of resource managers to protect and conserve migratory contingents within the larger Atlantic-wide population.

Coastal and offshore waters of the Gulf of Mexico (GoM) represent essential habitat for tarpon [7]. Based on collections of tarpon larvae (leptocephali) and early juveniles in the U.S. waters from Florida to Texas, adult tarpon spawn in coastal and offshore waters of the GoM in the late spring and early summer [7–9]. After a planktonic larval duration lasting approximately 20 to 50^+^ days, juveniles inhabit warm estuaries where predation is presumably lower and there is a readily available food supply. Juveniles remain in these inshore nurseries for several years before moving back into coastal waters as adults [7–9].

Once sexually mature (approximately 10 years of age and 120 cm fork length [FL]) [1, 10], tarpon spend the majority of their time in coastal and offshore waters in the GoM but are known to enter tidal passes and move back into estuaries [11, 12]. Similar to other regions, seasonal migrations displayed by tarpon in the GoM appear to be temperature dependent [13]. Luo et al. [2] showed that seasonal migrations in the fall and winter to lower latitudes were well developed for tarpon in the GoM, with different migratory patterns displayed by tarpon tagged east and west of the Mississippi River. Tarpon west of the river delta (hereafter western contingent) commonly crossed the Texas-Mexico border and overwintered in the southern GoM while tarpon east of the river delta (eastern contingent) moved to south Florida during the same period. Some evidence of eastern and western population structure was observed with nuclear DNA markers [14], supporting the premise that two contingents or subpopulations exist in the GoM. However, more recent studies using microsatellite DNA markers were unable detect genetic differentiation among tarpon in the GoM [15]. While these studies are valuable in identifying gene flow within the tarpon population, the level of mixing observed could be due to larval dispersal from currents and may not be directly related to adult tarpon traveling across regions.

Historically, tarpon in the western GoM supported substantial recreational tarpon fisheries between the 1920s and 1940s, with Port Aransas, Texas being referred to as the “Tarpon Capital of the World” [16]. This fishery collapsed in the 1960s and steep declines in tarpon landings were initially attributed to overharvesting [16]. However, corresponding reductions of juvenile tarpon landings led to speculation about recruitment failure due to the loss of nursery habitat or physiological stress related to cold snaps and cooler water temperatures off Texas being near their physiological limits [16]. Although mechanism(s) responsible for the decline of tarpon in this region are unknown, the primary impediment to managing the western contingent is the lack of data on the spatial distribution and movements of individuals. This information is needed to create appropriate regulations to manage tarpon along their seasonal migratory routes, which is inherently challenging because tarpon commonly cross management boundaries during their seasonal migrations and regulations often differ between states and countries [17].

The aim of this study was to characterize spatial distribution of juvenile tarpon and migratory pathways for adult tarpon from the northern GoM using historical catch data and conducting acoustic tagging, respectively. Gillnet surveys from Texas Parks and Wildlife Department (TPWD) were used to describe spatial distribution of tarpon in Texas bays and estuaries over the past four decades. Since TPWD catch data includes multiple environmental parameters, multivariable models were used to identify environmental conditions that define essential habitats of juvenile tarpon in this geographic region. To complement catch data, acoustic telemetry was used to characterize the movements of adult tarpon tagged both east and west of the Mississippi River Delta in the northern GoM. Griffin et al. [18, 19] previously utilized acoustic telemetry to successfully track tarpon movements in the southeastern United States and showed acoustic telemetry to be a valuable tool for tracking long-range movements of tarpon using collaborative acoustic networks. The use of acoustic telemetry afforded information on the migratory pathways of mature tarpon, which was then used to determine the degree of population mixing and/or straying between eastern and western migratory contingents. Acoustic telemetry data also allowed for insights on the timing of movements in recent years. The combination of catch data and electronic tagging used in this study will lead to an improved understanding of the habitat requirements and migratory pathways of tarpon in the northern GoM, which is critically needed by resource managers to assess the western contingent’s vulnerability and guide multi-state and multi-national conservation efforts to rebuild tarpon populations.

## Materials and methods

### TPWD gillnet catch data

**Fig 1:**
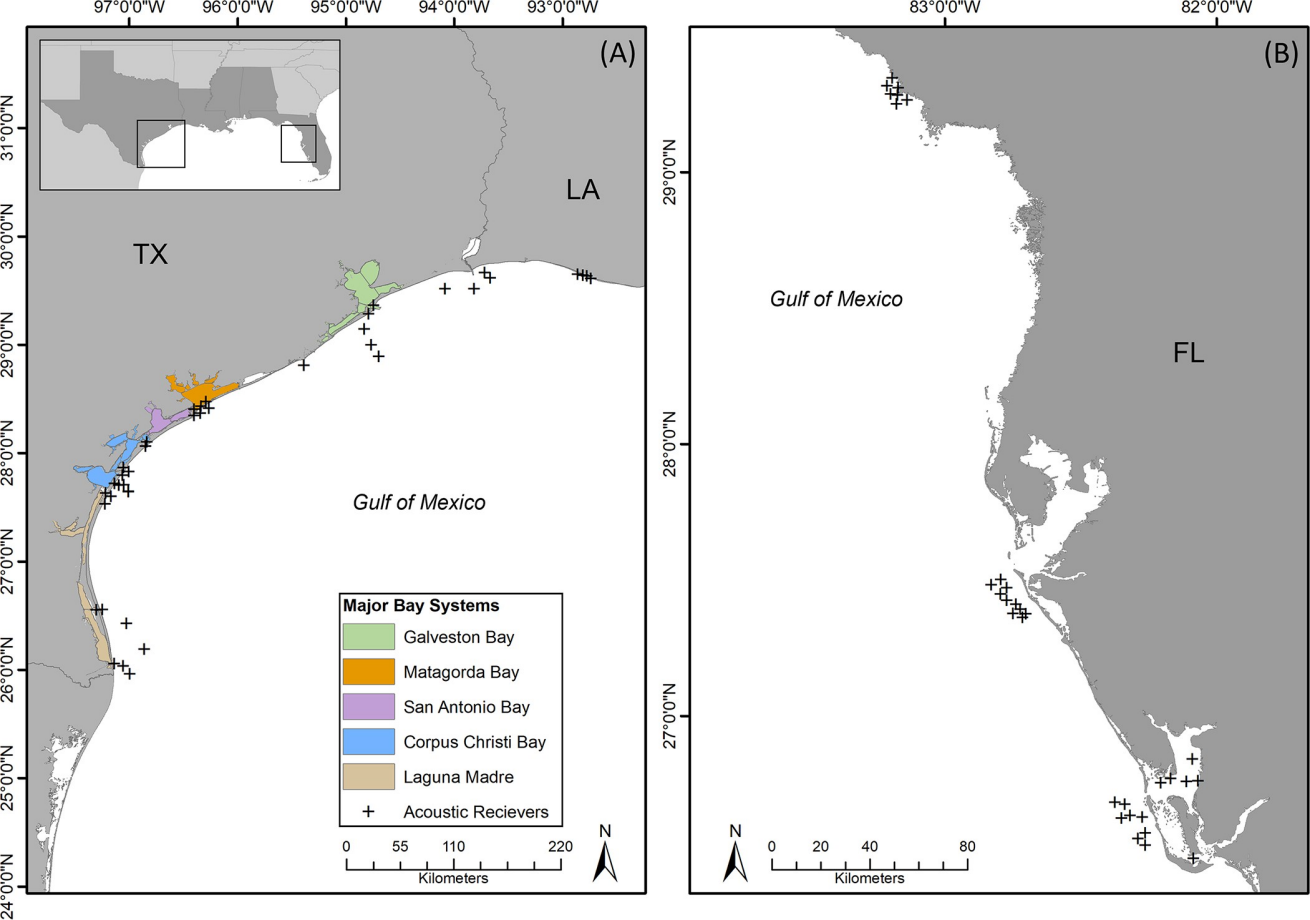
Bay systems sampled in gillnet surveys and the locations of coastal acoustic receivers. (A) Map showing the five major bay systems along the Texas coast where TPWD gillnet survey data was collected for this study along with the acoustic array located in coastal waters in the western Gulf of Mexico. (B) Map showing acoustic receivers in the iTAG network along the western Florida coast that detected tarpon acoustically tagged in this study.

Spatial and temporal patterns of juvenile and subadult tarpon in bay systems along the Texas coast were assessed using a long-term gillnet monitoring survey conducted by TPWD. Spring (April-June) and fall (September-November) gillnet surveys were conducted within 10 major sampling areas identified by TPWD (Sabine Lake, Galveston Bay, Cedar Lakes, East Matagorda Bay, Matagorda Bay, San Antonio Bay, Aransas Bay, Corpus Christi Bay, Upper Laguna Madre, and Lower Laguna Madre) from 1980 to 2018. Due to irregular sampling effort and low tarpon catch numbers, surveys from Sabine Lake, Cedar Lakes, and East Matagorda Bay were removed from the dataset. The remaining areas were summarized into five major bay systems; Galveston Bay, Matagorda Bay, San Antonio Bay, Corpus Christi Bay (comprised of Corpus Christi Bay and Aransas Bay), and Laguna Madre (comprised of upper and lower Laguna Madre; Fig 1). Texas bays are unique as they transition from hyposaline to hypersaline environments with decreasing latitudes. Minimum winter water temperatures also vary along the coast with warmer temperatures observed with decreasing latitudes. These bay systems were grouped according to their geographic proximity and expected similarity in environmental conditions. TPWD used a stratified clustered sampling design over the designated period, and set locations were randomly selected from a grid of one-minute latitude by one-second longitude cells. Gillnets were deployed within an hour of sunset and retrieved the next day within four hours of sunrise [20]. The monofilament gillnet used was 183 m in length and composed of four 45.7 m panels with differing stretched mesh sizes (76, 102, 127, and 152 mm). Gillnets were deployed perpendicular to the shoreline with the smaller mesh sizes closest to the shore. The date and time for each set was recorded along with environmental parameters including water temperature (°C), salinity (PSU), dissolved oxygen (mg l^−1^), and turbidity (NTU). When retrieved, elapsed soak time was recorded, and tarpon, along with other species, were identified to the species level, measured (FL in mm), and enumerated.

### Acoustic telemetry

Adult tarpon were tracked using an array of 39 Innovasea acoustic receivers (VR2W and VR2Tx) deployed in or near tidal passes and in coastal waters from the Texas-Louisiana border to the Texas-Mexico border in the western GoM. The array is comprised of a series of acoustic gates with receivers positioned in coastal water outside five of the major bay systems included in the TPWD gillnet surveys (Sabine, Galveston, Matagorda, Corpus Christi, and Lower Laguna Madre), and one area off the coast of western Louisiana (Fig 1). Receivers in the array were all deployed and remained in service for the duration of the study. At each location, receivers were deployed on a variety of structures including offshore rigs, pier pilings, submerged structures, or PVC pipe anchored into the sediment. In addition to receivers in coastal waters, receivers were positioned inside tidal passes (jetties) or in areas inside bays that are in close proximity to tidal passes to document potential estuarine-coastal movement of tarpon (Fig 1). Additionally, this study utilized other collaborative receiver networks in the eastern GoM through the iTAG program, and detection data from these additional arrays complemented data from the network of acoustic receivers in the western GoM, providing additional insights on the larger-scale movements of tarpon (Fig 1). Receivers were serviced approximately every four months, which included downloading data, removing biofouling on the external surface of receivers, and replacing batteries when required. All capture and release procedures were conducted in accordance with protocols approved by the Texas A&M University Institutional Animal Care and Use Committee (IACUC) #2021–0206 and #2020–0293. Adult tarpon (>120 cm FL) were tagged internally with Innovasea V16-4H (69 kHz) acoustic transmitters programmed with a 60–120 second random delay. This gave each transmitter an estimated battery life of approximately 1900 days. The high-power output of the V16 tag used can result in detection ranges up to 800 m; however, because water depth and physicochemical conditions in coastal area in the GoM vary it could be assumed that the range was likely lower. Tarpon were caught using conventional hook and line gear with artificial lures. Heavy tackle was used in order to reduce fight time. Once reeled up next to the tagging vessel, tarpon were positioned on the starboard side by securing the mouth at one end directing it towards the bow of the vessel and attaching a tail rope to the caudal peduncle at the opposite end. The vessel maintained a slow speed (approximately 0.5 to 0.7 m sec^-1^) to ensure that water passed through the mouth and over the gills of the tarpon and all fish remained in the water during the entire tagging process to increase survival rates. Tarpon were then rotated ventral side up putting the fish in a state of tonic immobility in an effort to reduce the stress of the individual [21, 22]. One to two scales were removed from the ventral side of the fish to allow for a small incision approximately 20 mm in length posterior and slightly dorsal of the pelvic fin. Incisions were slightly larger than the tag diameter size (16 mm), and Innovasea V16 transmitters were then inserted through the incision and into the peritoneal cavity of each tarpon. A suture was used to seal the surgery site using an Ethicon 4–0 monofilament suture in early deployments [18]; however, no sutures were used in the later surgeries to minimize handling and surgery time and in turn, reduce stress [23, 24]. Following the surgery, one scale was removed directly below the dorsal fin to allow for a conventional tag to be placed. This tag contained a unique identification number and contact information required to report any recaptured individuals. Tarpon were tagged both east and west of the Mississippi River Delta to have representation from both the eastern and western contingents.

### Data analysis

TPWD gillnet data was used to characterize trends in the presence and relative abundance of juvenile and subadult tarpon in five major bay systems along the Texas coast, which represented approximately 95% of the number of tarpon caught in gillnet surveys. Catch per unit effort (CPUE) of tarpon was generated from gillnet catches and standardized to soak time (number of tarpon per 1,000 hours). CPUE was then used as a metric of relative abundance to investigate potential regional and inter-decadal variation in tarpon catches in the western GoM Regional differences in CPUE were assessed using an analysis of variance (ANOVA) to identify significant differences among bay systems. In addition, tarpon presence/absence was used as dependent variables in estuary-specific generalized additive models (GAMs) parameterized with a suite of environmental parameters (independent variables) including season (spring and fall), decade, water temperature (°C), salinity (PSU), dissolved oxygen (mg l^−1^), and turbidity (NTU) to identify habitat requirements and environmental drivers that influence the distribution and abundance of tarpon. Models were made using the mgcv package in the statistical program R v.4.2.3 [25, 26]. The GAM modeling framework applied to gillnet data used a binomial distribution with a logit link and allowed for non-linear relationships that are common in ecology to be observed, and this modeling approach has been used successfully in other studies to determine fish-habitat relationships and identify key environmental drivers that influence habitat quality of many estuarine-dependent fishes [27, 28]. A manual backward stepwise selection procedure based on minimizing the Akaike Information Criterion (AIC) [29] using approximate p-values to help guide the selection process was used in order to select variables that would be included in final GAMs. Non-significant variables (p > 0.05) were removed one by one (based on largest p-value) in order to determine whether their exclusion improved the AIC, following Dance and Rooker [28, 30]. This was done until only significant variables were retained in the final model. Percent deviance explained (DE) was calculated to assess the overall fit of the model. Before the backward stepwise selection, collinearity between variables was identified using the VIF function in R [26]. If collinearity was detected between variables (VIF score >4), each was tested in the model to see which variable produced the better model fit (the lower AIC and higher DE) [26]. Once the final model was determined, ΔAIC and ΔDE for each of the remaining variables were calculated by removing each variable individually and comparing the difference in AIC and DE values to values from the original model. Both ΔAIC and ΔDE were used to evaluate the importance of each retained variable as seen in Zapp Sluis et al. [31]. A model for the entire coast (all bays combined) was examined; however, the ΔAIC and ΔDE were much lower compared to bay-specific models, which provided a better model fit. Therefore, only the bay-specific models were examined in this study.

Data generated from acoustic telemetry were used to assess the horizontal movement and migratory patterns of adult tarpon. Rate of movement (ROM) was calculated using known consecutive acoustic detections using ArcMap 10.7 and Geo Spatial Modeling Environment (GME) [32]. All consecutive detections or movements used in ROM calculations were within 150 days of the previous detection to prevent using two detections from potentially opposing seasonal migrations (e.g., northern versus southern migrations) in the same calculation. Consecutive detections also had to be greater than 5 km in distance in order to exclude small-scale movements within spatially limited acoustic arrays (e.g., acoustic gates). Rate of movement was calculated by dividing the distance of the movement (shortest possible in water route) by the time elapsed for specific intervals during the tracking sequence [33, 34]. Each ROM estimate was classified as “northern” or “southern” based on the direction (latitudinal change) of the observed track between consecutive detections. Rate of movement estimates for all tagged individuals were then averaged for each month and plotted to observe possible intra-annual trends in both ROM and directionality.

## Results

Overall, 407 juvenile tarpon (mean ± 1 SD: 67.9 ± 17.5 cm FL) were collected in TPWD gillnet surveys from 1980–2018 in the five major bay systems investigated (Galveston Bay [n = 18], Matagorda Bay [n = 53], San Antonio Bay [n = 33], Corpus Christi Bay [n = 111], and Laguna Madre [n = 192]). All tarpon collected in gillnet surveys used in this study were less than 120cm FL, and therefore assumed to be sexually immature. Over the 38 years of sampling, 23,830 gillnet sets were conducted and tarpon were present in 344 (1.4%) of those sets. Regional variation in CPUE and percent frequency of occurrence (%F) was observed (ANOVA: *P* < 0.001) with higher values found in the southern bay systems (Corpus Christi Bay CPUE [%F] = 1.20 [1.5%], Laguna Madre = 2.02 [2.2%]) relative to systems farther to the north (San Antonio Bay = 0.72 [0.9%], Matagorda Bay = 1.15 [1.3%], and Galveston Bay = 0.39 [0.5%].

**Fig 2:**
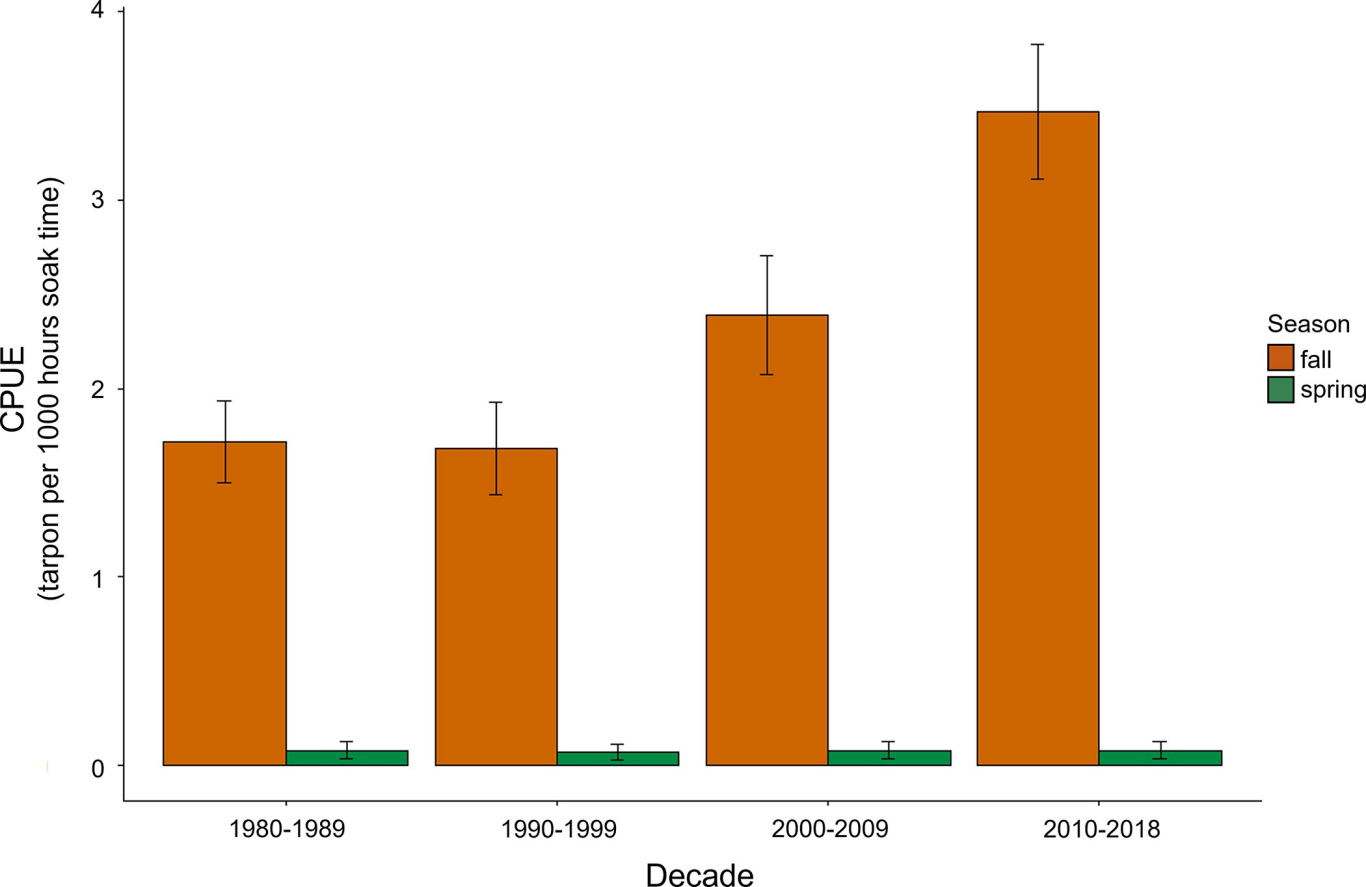
Seasonal catch per unit effort of tarpon found in gillnet surveys. Seasonal variation (spring and fall) in mean decadal CPUE (catch per 1,000 hrs. soak time) of tarpon collected in TPWD gillnet surveys from all five bay systems surveyed (Galveston Bay, Matagorda Bay, San Antonio Bay, Corpus Christi Bay, and Laguna Madre) pooled.

**Fig 3:**
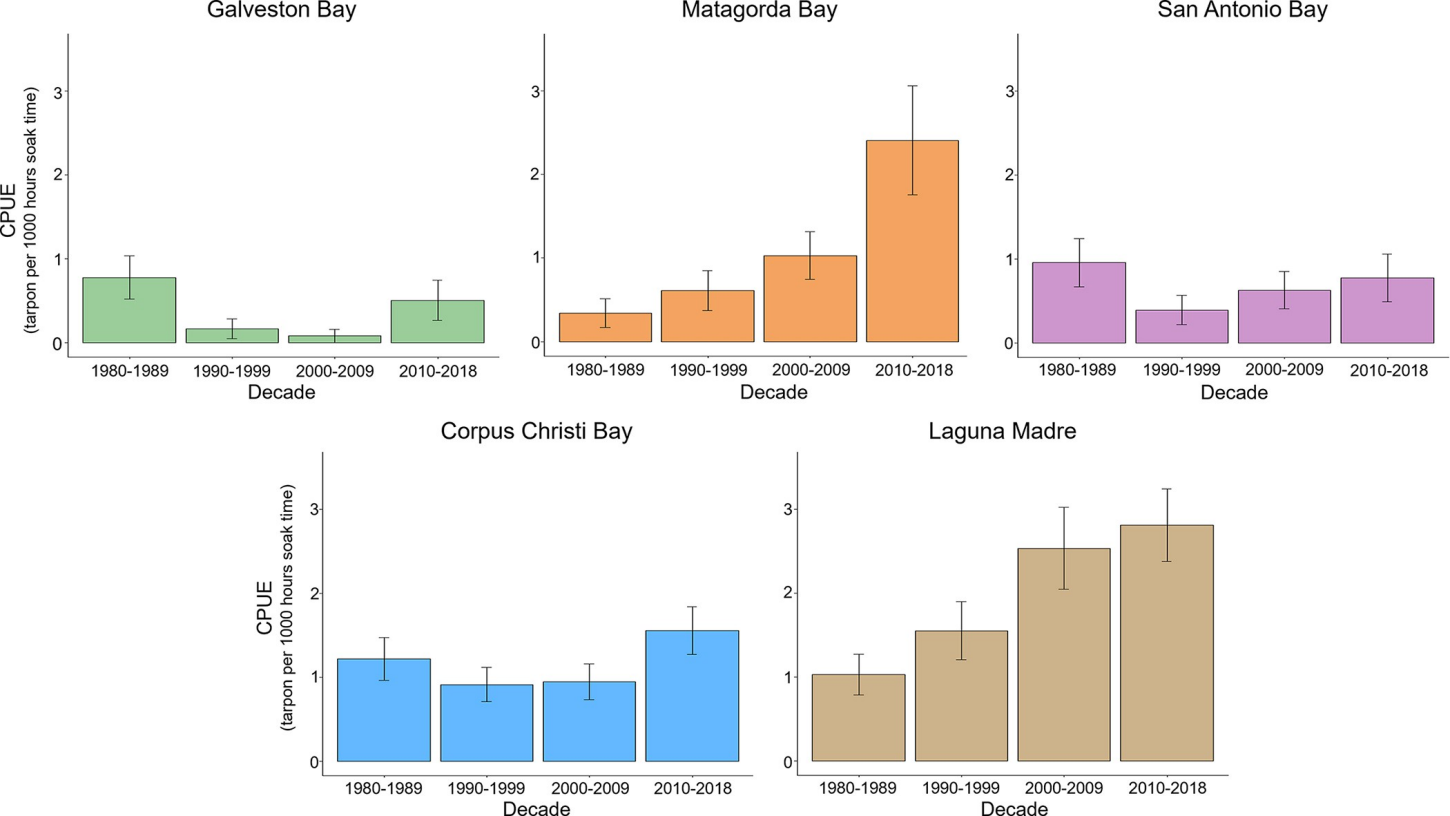
Mean decadal catch per unit of tarpon collected in gillnet surveys. Mean decadal CPUE (catch per 1,000 hrs. soak time) of tarpon in gillnet surveys for each of the five bay systems (Galveston Bay, Matagorda Bay, San Antonio Bay, Corpus Christi Bay, and Laguna Madre) from 1980 to 2018.

Pronounced seasonal and decadal trends in tarpon CPUE were observed along the Texas coast. Spring gillnet surveys had a mean decadal CPUE from 0.07 (1990–1999) to 0.08 (1980–1989) across all five major bay systems. Fall gillnet surveys resulted in mean decadal CPUE values two orders of magnitude higher than spring surveys, ranging from 1.68 (1990–1999) to 3.47 (2010–2018; Fig 2). Matagorda Bay and Laguna Madre were the only bay systems that experienced increasing CPUE of tarpon across each of the four decades investigated; nevertheless, CPUE for the most recent survey period (2010–2018) in all five bay systems was highest or second highest among the decades sampled (Fig 3).

### Fish-habitat models: Juvenile tarpon

#### Galveston bay

**Fig 4:**
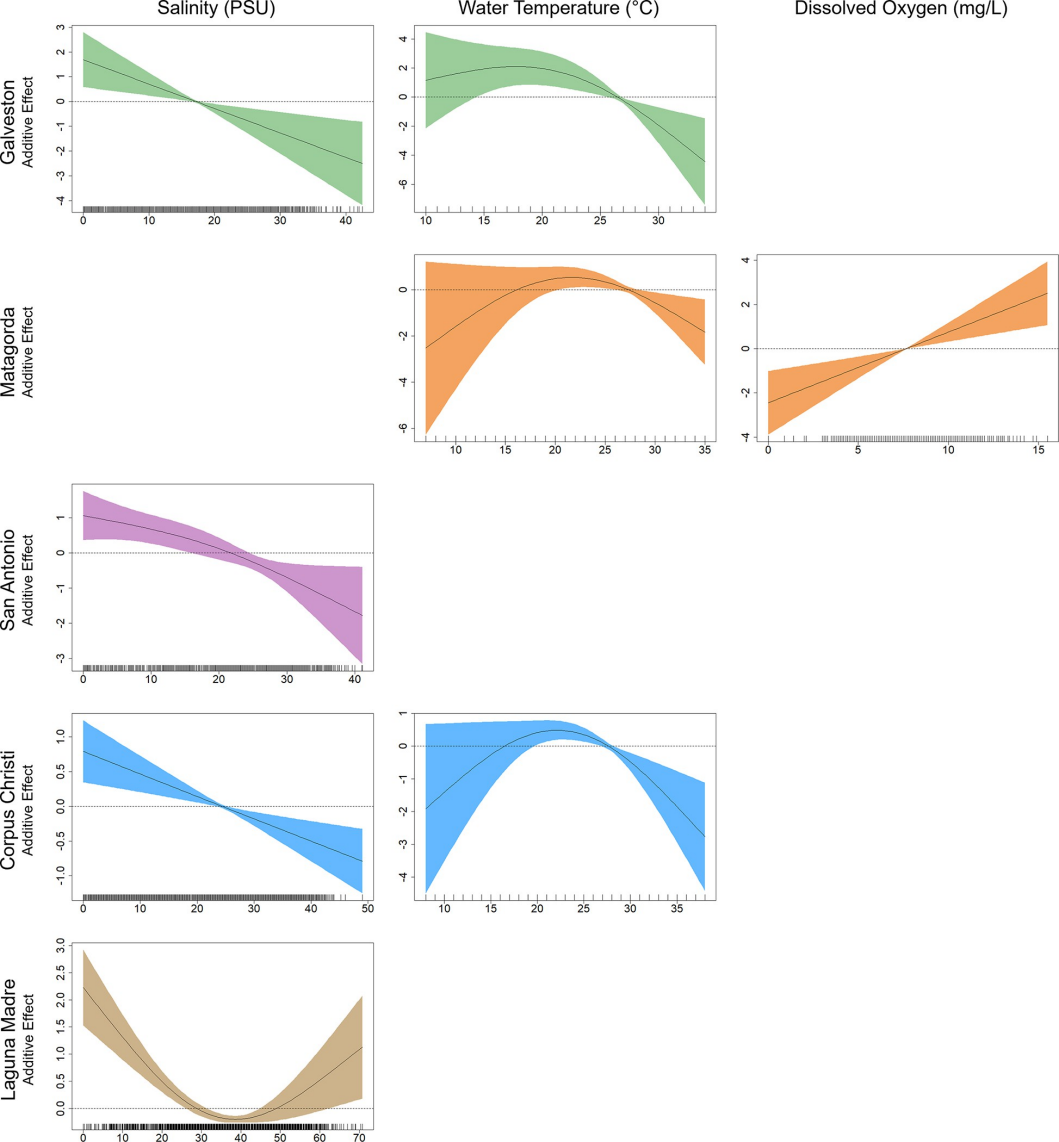
Response plots from generalized additive models for abiotic variables. Response plots showing additive effect of significant abiotic variables having an effect on tarpon presence from the final generalized additive models (GAMs) for each of the five major bay systems along the Texas coast. Plot contains salinity (PSU; left), temperature (°C; middle), and dissolved oxygen (mg l^−1^; right). Solid line represents smoothed values while shaded areas indicate 95% confidence intervals.

**Fig 5:**
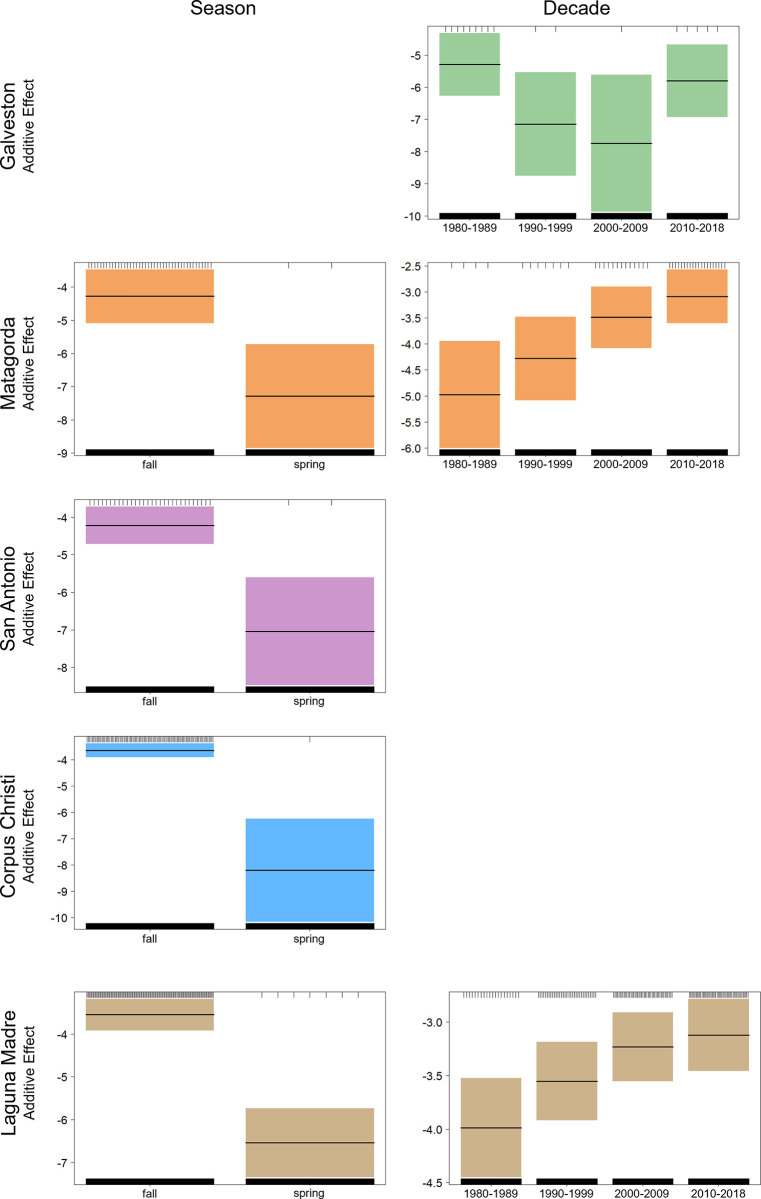
Response plots from generalized additive models for temporal variables. Response plots from the final generalized additive models (GAMs) for significant temporal variables having an effect on tarpon presence across each of the five major bay systems on the Texas coast. Plot depicts season (left) and decade (right). Shaded areas represent 95% confidence intervals.

The final GAM for tarpon presence in Galveston Bay (AIC = 188.18; DE = 18.6%) retained three variables: temperature (ΔAIC = 14.69; ΔDE = 8.7%), decade (ΔAIC = 6.64; ΔDE = 5.9%), and salinity (ΔAIC = 8.13; ΔDE = 4.8%). The presence of tarpon in Galveston Bay decreased with temperatures above 20°C, with the additive effect becoming negative at approximately 27°C. Response plots also indicated that tarpon presence in Galveston Bay declined as salinity increased, with the highest presence found in fresh and brackish waters (Fig 4). A significant inter-decadal trend was detected in the final model for Galveston Bay and the response plot showed that tarpon presence was highest in the earliest (1980–1989) and latest (2010–2018) survey periods (Fig 5).

#### Matagorda bay

The final GAM model for tarpon presence in Matagorda Bay (AIC = 404.75; DE = 17.3%) included four variables: season (ΔAIC = 39.73; ΔDE = 8.9%), decade (ΔAIC = 12.75; ΔDE = 4.0%), dissolved oxygen (ΔAIC = 9.01; ΔDE = 2.3%), and temperature (ΔAIC = 5.09; ΔDE = 1.8%). Seasonal trends showed that tarpon presence in Matagorda Bay was notably higher in fall gillnet surveys, and response plots also denoted a significant inter-decadal trend for Matagorda Bay with tarpon presence increasing each of the four decades surveyed (Fig 5). Response plots indicated that tarpon presence in Matagorda Bay was positively correlated with dissolved oxygen levels. The presence of tarpon in Matagorda Bay also increased with increasing water temperature up to approximately 22°C, with the additive effect becoming negative above 28°C (Fig 4).

#### San antonio bay

The final GAM for tarpon presence in San Antonio Bay (AIC = 325.61; DE = 12.2%) consisted of two variables: season (ΔAIC = 30.15; ΔDE = 8.9%) and salinity (ΔAIC = 11.66; ΔDE = 4.2%). Tarpon presence in San Antonio Bay show seasonal trends, with fall gillnet surveys having significantly higher tarpon presence compared to spring gillnet surveys (Fig 5).

Response plots also indicated that tarpon presence in San Antonio Bay was highest in fresh and brackish water, with tarpon presence declining as salinity increased (Fig 4).

#### Corpus christi bay

The final GAM for Corpus Christi Bay (AIC = 903.71; DE = 15.0%) retained three variables: season (ΔAIC = 115.85; ΔDE = 11.1%), temperature (ΔAIC = 11.23; ΔDE = 1.5%), and salinity (ΔAIC = 10.42; ΔDE = 1.2%). Seasonal trends for tarpon presence in Corpus Christi Bay were observed, with fall gillnets having markedly higher tarpon presence (Fig 5). Response plots for water temperature showed that tarpon presence in Corpus Christi Bay began to decline at 22°C, with the additive effect becoming negative as temperatures approached 28°C. Response plots also indicated that tarpon presence in Corpus Christi Bay was highest in fresh and brackish conditions and declined as salinity increased (Fig 4).

#### Laguna madre

The final GAM for tarpon presence in Laguna Madre (AIC = 1251.70; DE = 14.1%) included three variables: season (ΔAIC = 140.13; ΔDE = 9.8%), salinity (ΔAIC = 30.55; ΔDE = 2.4%), and decade (ΔAIC = 7.16; ΔDE = 1.0%). Significant seasonal trends were observed for tarpon presence in Laguna Madre with presence being higher for fall surveys (Fig 5). Response plots for tarpon presence in Laguna Madre indicated higher levels of tarpon presence in fresh and brackish water but also in hypersaline conditions greater than 50 PSU (Fig 4). Response plots also denoted a significant inter-decadal trend in the Laguna Madre with tarpon presence increasing in each of the four decades surveyed (Fig 5).

### Movement: Adult tarpon

From 2018 to 2022, 44 adult tarpon were tagged with V16 acoustic transmitters in coastal waters off Texas and Louisiana. Tagged individuals ranged in size from 127 to 196 cm FL (mean ± 1 SD: 167 ± 20 cm FL). The majority of tarpon were tagged off the coast of Texas (n = 40) from Matagorda Bay to Galveston Bay with a smaller number tagged in Louisiana east of the Mississippi River Delta (n = 4) to serve as an outgroup potentially comprised of individuals from the eastern migratory contingent. All 44 individuals were tagged between the months of June to October.

**Fig 6:**
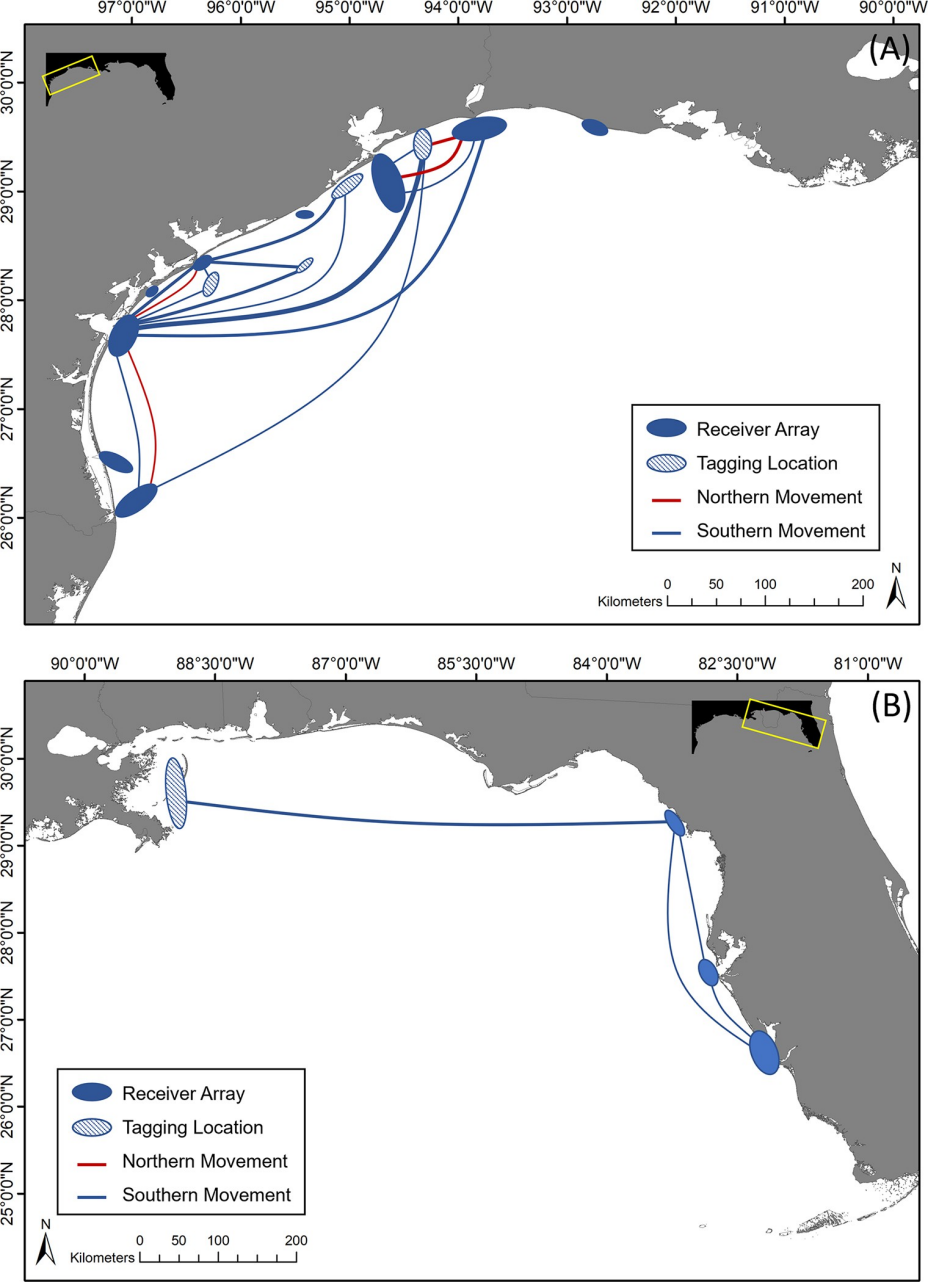
Tarpon movements within the Gulf of Mexico estimated from acoustic detections. Tarpon movement between coastal acoustic arrays in the western Gulf of Mexico (A) and eastern Gulf of Mexico (B) derived from acoustic transmitter detections. Red lines represent northern movements whereas southern movements are shown with blue lines with line thickness used to represent number of times the path of travel was utilized ranging from one (lightest) to three (heaviest). Dark blue ellipses on the map indicate sections of the coastal acoustic array. Light blue ellipses indicate locations that were used for tagging tarpon.

Overall, 18 of the 44 acoustically tagged individuals were detected (40.9%), generating 676 geographic position estimates. Of the individuals detected, 16 were tagged off the coast of Texas, with the remaining 2 being tagged off Louisiana east of the Mississippi River Delta. Detections were commonly recorded shortly after tagging events; however, 9 of the individuals detected produced tracking data for multiple years. None of the tagged tarpon were detected moving across the Mississippi River Delta from the initial tagging location. Tarpon tagged in Texas displayed both northern and southern migrations along the coast with multiple detections recorded at each of the regional acoustic receiver locations (i.e., gates) (Fig 6). Individuals tagged east of the Mississippi River Delta displayed large-scale southern movements between Louisiana and Florida (Fig 6).

**Fig 7:**
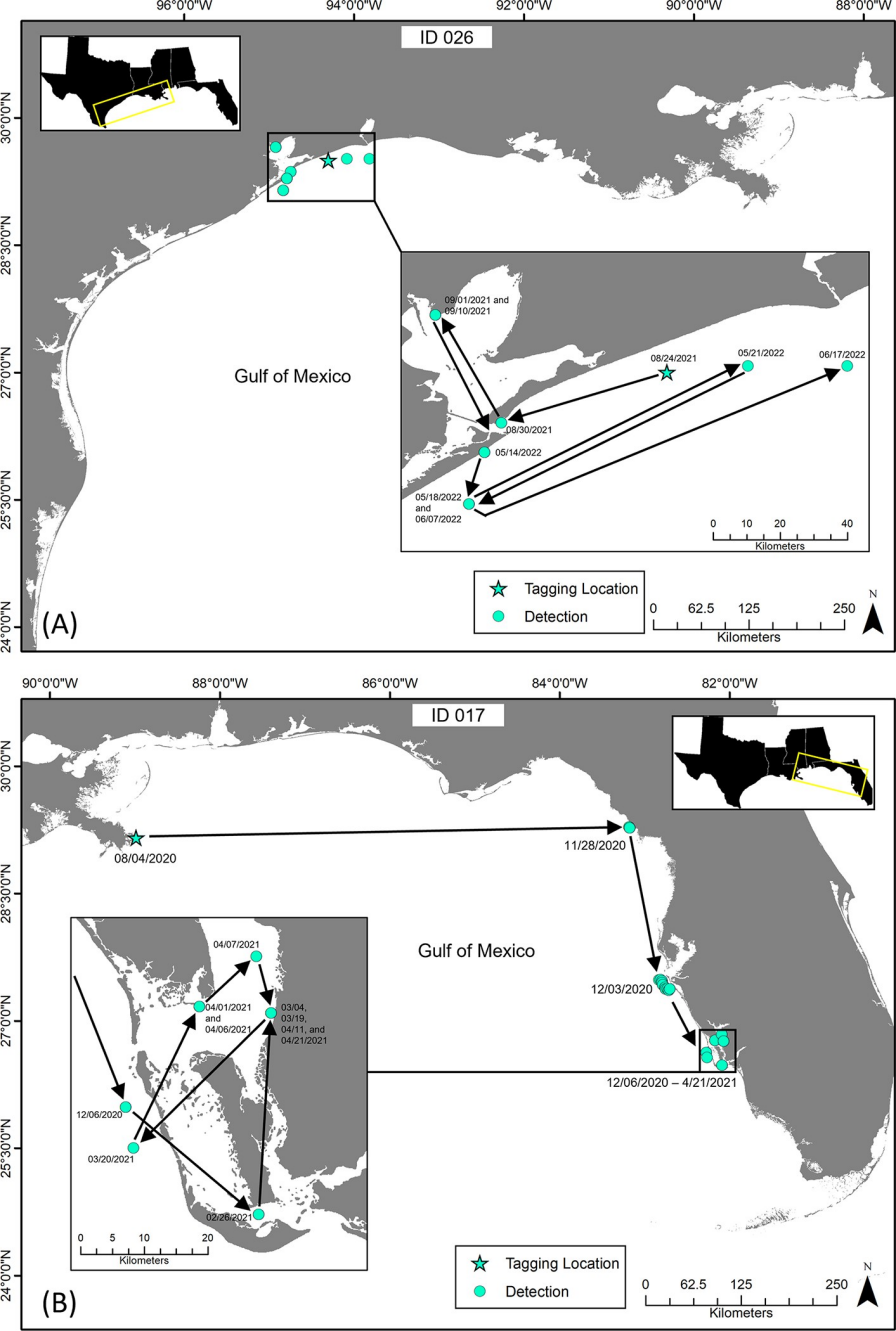
Individual tarpon movements, estimated from acoustic detections, demonstrating coastal-bay connectivity. Estimated straight-line tracks, derived from acoustic detections, of tagged tarpon showing examples of coastal-bay connectivity. Star symbols indicate tagging location of the individual and dots represent acoustic receivers that detected the tagged tarpon. Tarpon (ID 026; A) and tarpon (ID 017; B) show movement into bay systems for varying lengths of time during their migrations.

The majority of detections from tagged tarpon were recorded on receivers located in coastal waters, however, a subset of individuals (n = 9) displayed estuarine-coastal connectivity. These individuals were detected in bay systems in Texas and Florida. Time spent within the bays varied, reaching as high as approximately two months for some individuals. Tarpon (ID 026) tagged in coastal waters off northern Texas, moved into Galveston Bay six days after being tagged. The individual then spent 11 days in the bay, observed moving to the northern shoreline of the bay, before returning back to coastal waters (Fig 7). Tarpon (ID 017) was tagged east of the Mississippi River Delta, off the Louisiana coast. The individual was detected moving south along the Florida coast before moving into a bay system inshore of Boca Grande. It remained in the bay for 55 days before detections concluded (Fig 7).

**Fig 8:**
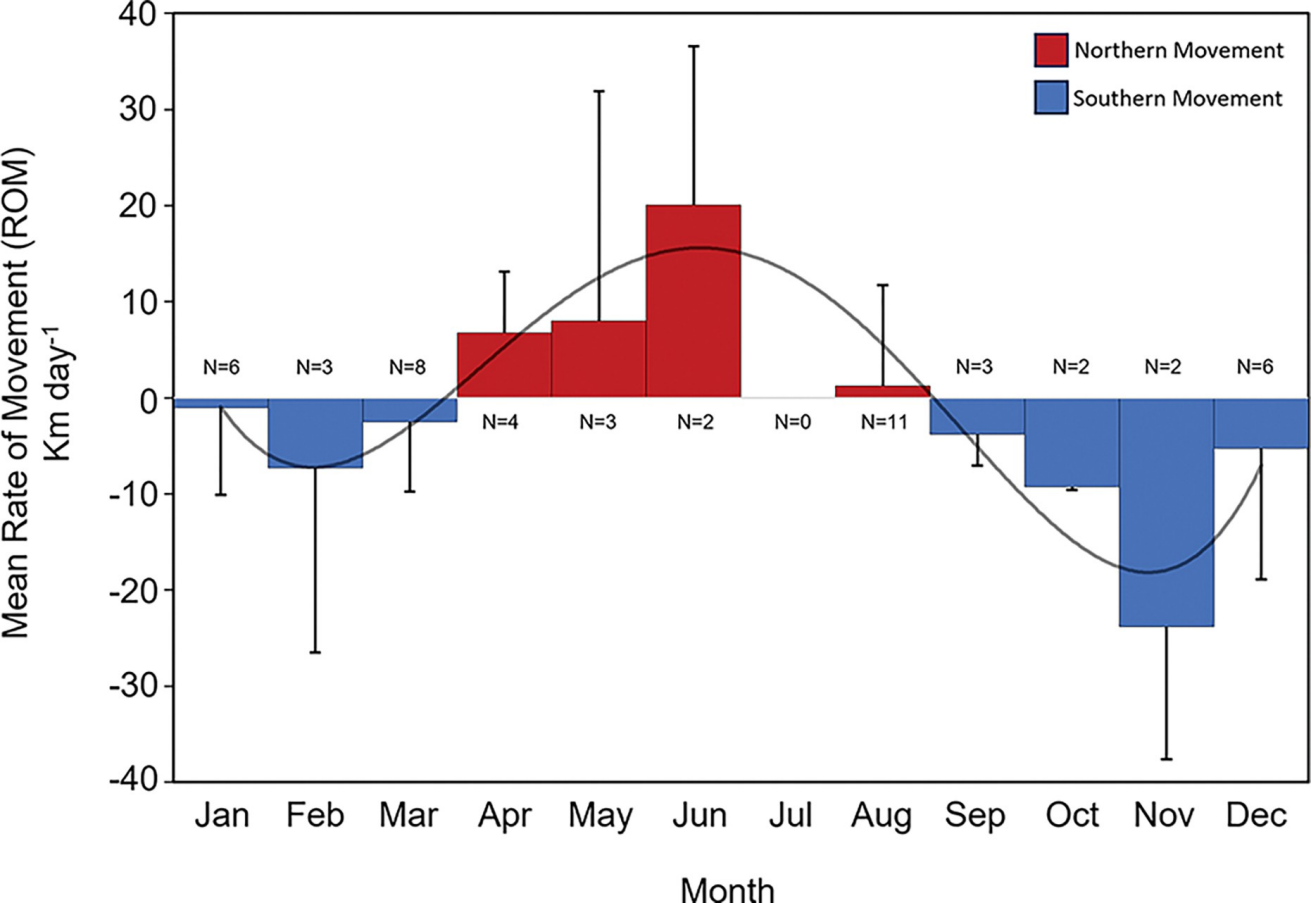
Mean monthly rate of movement from acoustically tagged tarpon in the Gulf of Mexico. Plot showing mean monthly rate of movement (ROM; km day^-1^) estimates of tarpon acoustically tagged in this study. Months with higher northern movements are shown with positive values and red columns, whereas months with southern movement trends are shown with negative values with blue columns. The number of movements used to calculate mean monthly ROM is listed for each month (N).

Rate of movement (ROM) estimates (distance between detections > 5km and elapsed time between detections < 150 days) ranged from 0.3 km day^-1^ to 35.6 km day^-1^. Of the 45 ROM estimates generated from 18 tagged tarpon, 26 were classified as southern movements and occurred from September to March (Fig 8). The month with the highest mean ROM for southern movements by tarpon was November (23.8 km day^-1^). The remaining 19 ROM estimates were classified as northern movements and occurred from April to August. Mean monthly ROM for northern movements by tarpon peaked in the month of June (20.0 km day^-1^; Fig 8).

## Discussion

Regional variation in the relative abundance (CPUE) and frequency of occurrence of tarpon was observed across estuaries in the western GoM, with the relative abundance from gillnet surveys being higher in more southern bay systems. This suggests that environmental conditions may be more favorable in southern bays than in bay systems to the north. The Texas coastline displays strong gradients with temperature increasing with decreasing latitude [28, 35]. Tarpon are susceptible to cold water temperatures and often do not survive freeze events [10, 36]. Mace et al. [36] observed juvenile tarpon had a mean minimum lethal temperature of 13.7°C, and winter water temperatures in northern Texas bays commonly approach or are below this minimum threshold. Thus, warmer southern bays generally experience higher winter temperatures and fewer freeze events, possibly leading to higher survival of juvenile tarpon [37]. Relative abundance of tarpon increased in all of the five major bay systems investigated since the 1990–1999 sampling period. Inter-decadal increases are evident in both northern and southern bay systems and most notable within the last decade of sampling (2010–2018) in this study. Increases in tarpon abundance across all Texas bay systems in the last decade of the survey may be associated with warming trends that have been noted as early as the 1970s and have accelerated over the last decade causing water temperatures to increase across this entire region [38, 39]. Rising mean annual temperatures are driven almost completely by an increasing trend in winter minimum temperatures [40]. The noticeable increase in tarpon abundance within the last decade (2010–2018) may be indicative of range expansion by tropical, warm-water species [41]. Fujiwara et al. [35] noted that warmer temperatures are capable of prompting poleward shifts in species ranges and prevalence, leading to increases in the presence of tropical, warm-water species in sub-tropical environments. Similarly, increased northern occupancy for tropical, warm-water species such as gray snapper (*Lutjanus griseus*) [40] and common snook (*Centropomus undecimalis*) [42] have been reported, and it is plausible to assume that tarpon may be experiencing similar expansions with warming conditions along the Texas coast. Moreover, prey items commonly consumed by tarpon such as Gulf menhaden (*Brevoortia patronus*), Atlantic croaker (*Micropogonias undulatus*), and white mullet (*Mugil curema*) have also experienced increased occupancy probabilities in Texas bays and the northern GoM as a whole due to climate change [35, 43]. Increases in prey availability and more suitable water temperatures experienced in the past few decades have likely produced more favorable environmental conditions for both juvenile tarpon, thus explaining observed increases in the relative abundance of tarpon across several bay systems. Another potential cause for the increasing abundance of juvenile tarpon in Texas bays during recent decades may be attributed to an increase of sexually mature adults, and the recovery of the GoM population could directly influence the abundance of juveniles recruiting to estuarine and coastal nurseries in Texas.

Seasonal shifts in the relative abundance of juvenile tarpon were markedly higher in the fall gillnet survey compared to the spring, suggesting that either overwintering mortality or movement to areas not sampled with TPWD gillnets may be occurring. Numerous studies have assessed residency times of juvenile tarpon in estuaries with peak residency periods occurring from July to December [44, 45]. In the present study, results show a similar trend with fall gillnet sampling season (September–November) coinciding with the period of peak residency observed in these previous studies. Winter mortality linked to sub-optimal temperatures (i.e., ‘winterkills’) has been observed for a variety of estuarine and marine fishes [45, 46], and thermal stress may be responsible for lower abundance of juvenile and subadult tarpon in the spring following a period of low winter temperatures in bays along the Texas coast. In addition to changes in winter survival, it is also possible that drops in water temperature may dictate the duration of estuarine habitat use by tarpon, especially in sub-tropical regions in the northern GoM. Temperature drops typically lead to fish searching for deeper waters for thermal refuge [36]. In response, tarpon in search of thermal refuge may move to deeper regions of the bay or in tidal passes during the winter that are not sampled by TPWD gillnets, or even move to channel systems within the bays or jetties. Assuming they remain in these areas into the spring, this may result in lower CPUE values during the spring survey.

Apart from temperature, regional variation in salinity is also pronounced in Texas with elevated salinity typical of more southern bay systems. While tarpon can tolerate a wide range of salinity, early life stages (larvae) are often collected in waters of higher salinity (~30–40 PSU) [47]. Cofill-Rivera et al. [37] found juvenile tarpon were able to tolerate lower water temperatures at higher salinities (≥30 PSU), resulting in higher overwintering survival. As a result, higher salinity in southern bay systems may lead to increased survival of early life stages of tarpon inhabiting these regions, subsequently leading to higher numbers of juveniles. While the physicochemical properties of southern bay systems may elevate habitat quality for early life stages of tarpon, the higher relative abundance of tarpon in these bay systems may be related to their proximity to spawning grounds. Previous studies identified potential tarpon spawning locations off the coast of south Texas from Matagorda to Laguna Madre [2]. The proximity of bay systems in the south to putative spawning grounds may result in higher larval supply and recruitment, and therefore influence regional patterns of abundance. Future use of larvae drift models could help determine regional estimates of larval supply.

Similar to regional CPUE patterns observed across the bay systems sampled in Texas, GAMs indicated that salinity and water temperature were important environmental factors affecting tarpon presence within most of the bay systems investigated. Models developed for Galveston Bay, San Antonio Bay, Corpus Christi Bay, and Laguna Madre all showed increased tarpon presence in fresh and brackish water with presence decreasing as salinity increased to approximately 20 PSU. Although the Laguna Madre model showed a negative effect of salinity on tarpon presence starting at approximately 20 PSU, the response plot indicated an increase in tarpon presence at salinities greater than 50 PSU. This result is likely unique to the Laguna Madre since this bay system often becomes hypersaline with a mean salinity of approximately 31 PSU and salinities ranging up to 60–100 PSU at times of low rainfall levels [48]. Previous studies have observed tarpon tolerating a wide range of salinities, from fresh to hypersaline environments throughout their life history [2, 6, 7]; however, juveniles are often found in rivers, bays, and estuaries, highlighting the importance of fresh and brackish water habitats during the first year(s) of life [6, 17, 45]. GAMs also revealed that tarpon presence generally increased at temperatures between 20–25°C. This finding is in accord with the preferred temperature range of tarpon (20–26°C) reported in several other studies that document this preferred range in both juvenile and adult tarpon [5, 6, 13]. While salinity and temperature were influential in explaining tarpon presence, dissolved oxygen was not deemed to be an important environmental factor in the majority of models, even though this factor is commonly found to restrict the distribution of many estuarine finfish species [49, 50]. The limited influence of dissolved oxygen in final models is likely because tarpon can gulp air to facilitate respiration in areas of the bay with low dissolved oxygen levels [51, 52].

Collectively, migrations observed for acoustically tagged tarpon support the hypothesis of two distinct migratory contingents occurring in the GoM, which occur east and west of the Mississippi River Delta. Similarly, previous research suggested the presence of both eastern and western migration routes for tarpon in the GoM based on findings from satellite tracking [2]. In the present study, tarpon tagged east and west of the Mississippi River Delta often moved significant distances, but no individuals were observed crossing the delta. Seasonal migrations by tarpon to waters proximal to the Mississippi River Delta have been previously reported, and movement into this area is ostensibly associated with spawning and foraging behaviors [9, 45, 53]. Nutrient loading from the Mississippi River enhances primary and secondary productivity, which is potentially the reason that tarpon and other large predators move into this region in the summer and fall [2, 53, 54]. However, the inflow from the Mississippi River also creates strong gradients in physicochemical conditions that may serve as a barrier and restrict the movements of tarpon across this feature, ultimately limiting the exchange of tarpon on each side of the delta [55, 56]. Patterson & Cowan [57] noted similar trends when tagging red snapper (*Lutjanus campechanus*) where the vast majority of individuals tagged east of the Mississippi River Delta moved east and only one individual moving west across this feature, supporting the premise that the delta may restrict movements.

Movements of individual tarpon observed in this study from acoustic detections provided insights into habitat use and migration pathways of both eastern and western contingents. Some individuals were detected on the same receivers almost exactly a year apart from previous detections, suggesting fidelity and return migrations at approximately the same time of year. Griffin et al. [19] observed repeatability in tarpon movements at an individual level to be high, identifying photoperiod to be an important cue for migration. Many other marine species follow common migration pathways with geographic routes that are repeated at similar times each year, and many of these recurring migrations are associated with spawning and foraging [19, 58, 59]. Kurth et al. [60] found evidence of migratory fidelity for tarpon, using stable isotope analysis, with individuals collected off Louisiana returning to the same coastal system each year. In addition to displaying common migration pathways in coastal waters, several tarpon tagged in this study were also observed moving from coastal waters back into estuaries, indicating a relatively high degree of estuarine-coastal connectivity for adult tarpon. Luo et al. [2] found similar results using satellite tags with over 50% of the individuals tagged in coastal waters occasionally moving into estuaries and rivers. A possible benefit to returning to estuaries by adult tarpon could be to reduce parasites [61]. Another potential motivation for adult tarpon moving into estuarine habitats may be associated with enhanced prey availability since primary and secondary productivity is often higher in these bay systems [12].

Rate of movement estimates of tarpon were used to quantify the timing and directionality of observed movements. Mean monthly ROM estimates indicated that southern migrations started in September and lasted through March with the highest mean monthly ROM estimates occurring in November. Northern movements that met the time and distance threshold used for this study were limited. This is likely due to low sample size, especially in the eastern GoM. Movements farther offshore, outside the extent of the arrays, could also account for the limited northern movements observed. Migrations are commonly driven by pronounced changes in environmental conditions [62], and November typically brings some of the first major cold fronts to the Texas coast. These fronts often affect tidal currents, water temperature, and water levels in estuarine and coastal systems, and these changes may initiate seasonal migrations of tarpon to more southern locations, subsequently leading to the observed increases in ROM estimates. Because tarpon are generally regarded as tropical, warm-water species, pronounced drops in water temperature appear to trigger the initiation of southward migrations. Similarly, other species are known to migrate seasonally to reduce environmental variability experienced throughout the year [62]. Numerous studies have found tarpon movements to be closely related to the 26°C isotherm, indicating tarpon migrate north and south to inhabit preferred or optimal water temperatures [2, 5, 6]. Apart from maintaining optimal conditions for adults, tarpon migrations may also be associated with spawning adults moving into areas that may serve as highly suitable habitats for early life stages of tarpon, which has been observed for other highly migratory species that return to warm, productive waters, such as those found in the northern GoM, to spawn [63–65]. Crabtree et al. [7] used collections of tarpon larvae (leptocephali) to estimate the general location and timing of spawning for tarpon, with the latter occurring from late spring to late summer in Florida. In the present study, acoustically tagged tarpon were observed moving north along the coast of Texas from April through August, peaking in June, and these return migrations coincide with the presumed spawning season of tarpon and were similar to findings from satellite tagged tarpon in the GoM [2]. As mentioned previously, Luo et al. [2] identified potential spawning locations in the northern GoM including an area from Matagorda Bay to the Lower Laguna Madre along the coast of Texas; however, the region north of Matagorda to the Mississippi River Delta was not deemed to be an important spawning area for tarpon. Although the number of detected individuals west (n = 14) and east (n = 2) of the Mississippi River Delta were low, based on detection data of tarpon during the presumed spawning periods mentioned above, the presence of sexually mature tarpon in the northwestern GoM during this time frame may indicate that this region represents a new spawning area not previously known for tarpon. The collection of tarpon larvae and juveniles from northern bay systems in Texas (e.g., Galveston Bay, Sabine Lake) supports the hypothesis of spawning extending into northern areas in the northwestern GoM (TPWD, unpublished data). Nevertheless, future research using biophysical transport models to estimate larval drift for tarpon in the northern GoM would provide more definitive insight on the location of spawning grounds in the GoM.

Results from this study suggest that Texas estuaries represent essential habitat for both juvenile and adult tarpon. Current warming trends will play a significant role in determining the distribution and abundance of tarpon in the GoM, with relative numbers of tarpon likely increasing over time to higher latitudinal zones in Texas as individuals expand their northern range. Expansion of suitable habitat for juvenile tarpon along the Texas coast likely drives the recovery of adult tarpon observed off Texas, which also promotes higher recruitment back into the bays. Movement data observed using acoustic telemetry provided more insight on the timing and migration pathways of tarpon, while also providing evidence for the presence of two distinct migratory contingents (east and west) divided at the Mississippi River Delta. Increased tagging effort on both the eastern and western sides of the delta are needed to fully understand the nature of the population structure and potential exchange between eastern and western contingents. Telemetry data from this study also demonstrated that tarpon move across state borders. Further, the lack of detections in the winter after moving into coastal waters off Brownsville, Texas near the international border probably signifies that a fraction of the tarpon cross into territorial waters of Mexico. The movement of tarpon across state and federal borders where regulatory measures often differ indicates the strong need for cooperative management to sustainably manage the tarpon in the GoM. While cooperative management could take the form of more consistent bag limits and minimum size restrictions across borders, the tarpon fishery is largely a catch and release fishery. Thus, implementing regulations that promote and enforce safe handling practices, such as those in Florida, would likely have a greater impact on the recovery and conservation of tarpon throughout its range in the GoM.
